# Development of a Behavior Change Intervention to Encourage Timely Cancer Symptom Presentation Among People Living in Deprived Communities Using the Behavior Change Wheel

**DOI:** 10.1007/s12160-016-9849-x

**Published:** 2017-12-13

**Authors:** Stephanie Smits, Grace McCutchan, Fiona Wood, Adrian Edwards, Ian Lewis, Michael Robling, Shantini Paranjothy, Ben Carter, Julia Townson, Kate Brain

**Affiliations:** 1 Division of Population Medicine, School of Medicine, Cardiff University, Neuadd Meirionnydd, Heath Park, Cardiff, UK; 2 Tenovus Cancer Care, Cardiff, UK; 3 South East Wales Trials Unit, Centre for Trials Research, Cardiff University, Cardiff, UK

**Keywords:** Cancer, Complex intervention, Qualitative, Behavior change, Inequality, Symptompresentation

## Abstract

**Background:**

Targeted public awareness interventions are needed to improve earlier cancer diagnosis and reduce socioeconomic inequalities in cancer outcomes. The health check (intervention) is a touchscreen questionnaire delivered by trained lay advisors that aims to raise awareness of cancer symptoms and risk factors and encourage timely help seeking

**Purpose:**

This study aimed to apply the Behavior Change Wheel to intervention refinement by identifying barriers and facilitators to timely symptom presentation among people living in socioeconomically deprived communities

**Methods:**

Primary data (six focus groups with health professionals, community partners and public) and secondary data (systematic review of barriers and facilitators to cancer symptom presentation) were mapped iteratively to the Behavior Change Wheel.

**Results:**

Barriers and facilitators were identified from the systematic review and focus groups comprising 14 members of the public aged over 40, 14 community partners, and 14 healthcare professionals. Barriers included poor symptom knowledge and lack of motivation to engage in preventive or proactive behaviors. Facilitators included cues/prompts to action, general practitioner preparedness to listen, and social networks. The following behavior change techniques were selected to address identified barriers and facilitators: information about health consequences, prompts/cues, credible sources, restricting physical and social environment, social support, goal setting, and action planning.

**Conclusions:**

The Behavior ChangeWheel triangulated findings from primary and secondary data sources. An intervention combining education and enablement could encourage timely symptom presentation to primary care among people living in socioeconomically deprived communities. Social encouragement and support is needed to increase symptom knowledge, challenge negative cancer beliefs, and prompt decisions to engage with the healthcare system.

## Teaser

A targeted intervention combining education and enablement could encourage timely cancer symptom presentation among people living in socioeconomically deprived communities.

## Background

Socioeconomic inequalities in stage at cancer diagnosis and survival are well documented [1–3], partly due to delayed symptom presentation among socioeconomically deprived populations [4, 5], as well as reported links to low knowledge or awareness of cancer symptoms, negative beliefs about cancer, and low health literacy [6, 7]. The revised National Awareness and Early Diagnosis Initiative (NAEDI) pathway includes socioeconomic status, age, sex, and ethnicity as influences on cancer survival and premature mortality in the UK. The NAEDI pathway (supplementary file 1) hypothesizes that these factors influence presentation to primary care in terms of low public awareness, barriers to help seeking, and negative beliefs about cancer, which in turn influence primary and secondary care delays [8]. Improved cancer survival outcomes could therefore be achieved through early detection and diagnosis [9, 10].

Barriers to early symptom presentation include fatalism and denial [11]; fear of diagnosis, fear of treatment, and fear of dying [12]; and misinterpretation of symptom seriousness [13]. Other barriers include concerns about wasting the doctor’s time [14], difficulties making appointments [6, 14, 15], and lack of continuity in primary healthcare [16]. A systematic review of the influences of awareness and beliefs on symptom presentation reported that the combination of fearful and fatalistic beliefs is associated with longer presentation times in lower socioeconomically deprived populations [12]. Empirical evidence about social influences on help seeking and symptom presentation suggests that the social context in which perceptions about illness and health systems are formed are important considerations that are often overlooked in research [17].

Evidence-based initiatives that target public awareness and reduce barriers to early cancer diagnosis among people living in socioeconomically deprived communities are needed to improve cancer outcomes and reduce inequalities [18, 19]. Targeted community-based interventions that attempt to use social norms and influences show promise, by promoting positive attitudes and increasing motivation to present early while challenging automatic negative associations of cancer as a death sentence [11]. To date, interventions designed to increase cancer awareness and encourage help seeking have not been targeted at people living in socioeconomically deprived communities. The Medical Research Council guidance for the development and evaluation of complex interventions emphasizes the importance of developing a theoretical understanding of the area in question and embedding of interventions in local needs, preferences, and priorities to ensure that the intervention fits the needs of the community [20]. Tenovus Cancer Care (a cancer charity based in Wales, UK) have developed an innovative community outreach intervention as a potential way of engaging people in socioeconomically deprived communities to become more aware of cancer symptoms. The health check is a touchscreen questionnaire delivered face-to-face by a trained lay advisor that aims to raise awareness of cancer risk factors and symptoms and encourages people to seek medical help in the presence of symptoms. To date, 3898 original health checks have been completed across 147 events. However, due to funding and capacity issues, these were not evaluated to measure effectiveness. A study of effectiveness, including follow-up assessment, is needed in order to provide evidence before the intervention can be implemented.

Interventions designed to increase cancer symptom awareness and timely help seeking are more likely to be effective not only if they are developed in partnership with local stakeholder groups but if they are also informed by a theoretical understanding of how cancer awareness interacts with cancer attitudes and beliefs in influencing help-seeking intentions and behaviors in socioeconomically deprived communities [21]. We sought to refine the content of the health check and identify essential elements to include based on the Behavior Change Wheel [22], a model of behavior change that is based on an integrated framework of existing theories [22].

**Fig. 1:**
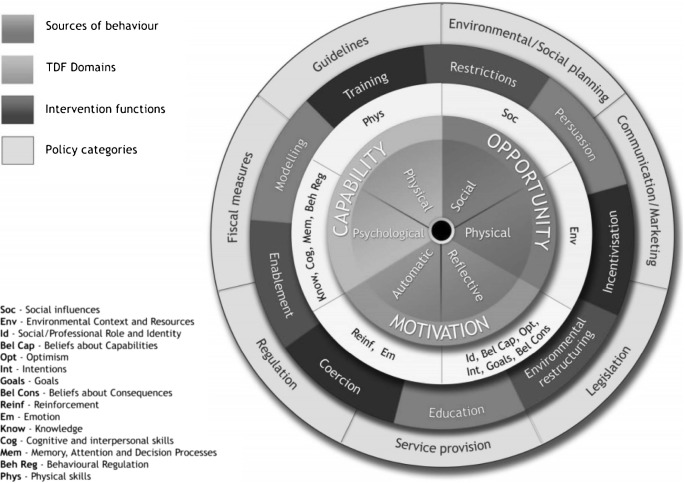
Behavior Change Wheel, which highlights the COM-B model (green), TDF (yellow), and intervention functions (red) [25]

The Behavior Change Wheel is a multiphase process guide for developing complex behavior change interventions, which identifies sources of behavior that could be targeted in interventions (see Fig. 1). The Behavior Change Wheel comprises the COM-B model and is supported by the Theoretical Domains Framework (TDF), the intervention functions mapping matrices, and a taxonomy of behavior change techniques [23, 24]. The COM-B model describes how changing behavior (B) is a result of changing one or more components of psychological and/or physical capability (C), social and physical opportunities available (O), and automatic and reflective motivation (M). The TDF provides a more granular level of understanding and consists of domains that can be condensed to fit the three components of the COM-B model, as follows: capability (knowledge, cognitive and interpersonal skills, memory, attention and decision processes and behavioral regulation, physical skills), opportunity (social influences, environmental context, and resources), and motivation (reinforcement, emotions, social/professional role and identity, beliefs about capabilities, beliefs about consequences, goals, and intentions) [25]. Capability and opportunity can influence motivation while behavior can influence capability, opportunity, and motivation [26]. The COM-B model and TDF can be used together to identify what needs to change in order to bring about the target behavior. Intervention functions are then considered (see Fig. 1). These are broad categories aligned to the COM-B Model through which an intervention can change behavior [24] and include education, persuasion, incentivisation, coercion, training, restriction, environmental restructuring, modeling, and enablement [24]. The behavior change techniques taxonomy (v1) is a separate tool that is linked to the Behavior Change Wheel and assists decision-making about specific behavior change techniques (BCTs) to include in the intervention, based on the identified intervention functions [23]. BCTs are active ingredients that will bring about change and are selected to deliver the intervention functions [23].

The aim of the study was to refine the content and delivery of the cancer awareness intervention (health check) aimed at encouraging timely symptom presentation among people aged 40 years and over living in socioeconomically deprived communities, using the Behavior Change Wheel as a guide.

Specific objectives were to (1) gather evidence from primary (focus groups) and secondary (systematic review) data sources to identify barriers and facilitators to symptom presentation in people living in socioeconomically deprived communities and determine which barriers and facilitators need to be addressed in the health check, (2) identify the type of intervention functions required to bring about the change, and (3) identify specific BCTs aligned to the purpose of the health check.

## Method

### The Intervention

The health check consists of 30 questions in three domains: your history, your lifestyle, and your health (see supplementary file 2 for full list of questions). Questionnaire responses are used to provide individualized advice and signposting to primary care and other relevant services. Responses for each question are given in a traffic light system, with “green” indicating results where no change is suggested, “amber” indicating an area where change or signposting could be considered, and “red” results indicating that action should be taken. Text-accompanying results provide information and guidance based on individual results. The health check was initially developed in 2009 and used exclusively on-board a mobile cancer support unit. In 2011, a new online version was developed to enable delivery in multiple locations simultaneously. Existing content was developed by a group of Tenovus Cancer Care staff across several departments (research, health and well-being, and the cancer support team).

The health check has previously been delivered by Tenovus in a variety of community settings (mobile units, GP practices, and public areas such as community centers and workplaces) in deprived areas of South and North Wales. The current study provided an opportunity to formally refine, develop, and evaluate the health check in a research context. Theoretically driven changes were identified using the Behavior Change Wheel, with empirically driven changes made based on the latest clinical guidelines for the referral and recognition of suspected cancer in the UK (https://www.nice.org.uk/guidance/ng12?unlid=49677604320162173631).

A process of mapping the health check to the Behavior Change Wheel was undertaken in three stages separately by SS, KB, and GM in order to refine and develop health check content. Discrepancies in mapping or coding were discussed and resolved. The methods used to address each of study objectives 1 to 3 are described below.

1. Identification of barriers and facilitators to timely symptom presentation among people living in socioeconomically deprived communities

The aim of the intervention is to encourage timely presentation to primary care in people who have symptoms indicative of possible cancer. Primary (focus groups) and secondary (systematic review) evidence sources were used to identify barriers and facilitators to the target behavior.

## Systematic Review

Findings from a systematic review of barriers and facilitators to early symptom presentation (13) were mapped on to the COM-B model and TDF [22] in order to understand the target behavior in terms of capability, opportunity, and motivation. The systematic review was conducted on studies of actual or anticipated symptom presentation across all tumor sites and highlighted that poor knowledge, fearful and fatalistic beliefs about cancer, and emotional barriers lead to later presentation among lower socioeconomic groups [12]. The methods for the systematic review are described in full elsewhere [12].

Barriers and facilitators identified in the 60 papers included in the review were extracted for use in the current study. Extracted barriers and facilitators were mapped on to the COM-B/TDF independently by KB, SS, and GM. The mapping process was facilitated by an Excel spreadsheet with COM-B components and related TDF domains in separate columns, and each barriers and facilitators added as a row. KB, SS, and GM independently coded each barrier and facilitator to the COM-B and TDF. Any discrepancies in coding were resolved through discussion.

In order to streamline the number of barrier and facilitator that could feasibly be targeted in the intervention, all barriers and facilitators were categorized as modifiable or non-modifiable separately by SS, KB, and GM [24]. This was done by considering whether each individual barrier and facilitator was within the scope of the intervention. For example, “continuity of care” was not considered to be modifiable because the intervention is targeted at the level of the individual rather than the medical system. Again, any discrepancies were resolved through discussion.

## Focus Groups

Focus groups were undertaken in Communities First areas in Wales to gain local stakeholders’ views regarding cancer awareness and health check content. Communities First is a Welsh Government community program focused on tackling poverty that supports the most disadvantaged people in the most deprived areas of Wales with the aim of contributing to the alleviation of persistent poverty. Focus group topic guides were constructed based on barriers and facilitators identified during the COM-B/TDF mapping process of the systematic review and included questions related to capability, opportunity, and motivation. Topics included the importance of health for people in the local community, what people in the community would do if they had a potential symptom of cancer, barriers and facilitator to early symptom presentation, perceived benefits of the intervention, and intervention delivery (supplementary file 3). Ethical approval was received from the National Research Ethics Service (NRES) Research Ethics Committee (REC reference no 14/NW/1104), and all participants provided written informed consent.

## Sample

Six focus groups were conducted in two health boards (Aneurin Bevan University Health Board and Cwm Taf University Health Board). Separate focus groups were conducted with healthcare professionals (general practitioners, practice nurses, practice managers, community pharmacists and public health consultants), community partners (housing association workers, Communities First workers, and other community-based workers), and members of the general public (males and females aged over 40 living in Communities First areas). Separate focus groups were held in these groupings to encourage attendance and facilitate relaxed and open discussion. Participants were reimbursed for their time in attending the focus groups. Opportunistic sampling was used with local study collaborators and snowballing to identify participants. A member of the project team within each health board acted as local study collaborator and aided recruitment by providing stakeholder lists of healthcare professionals, public health specialists, and community partners. The public focus groups were conducted last so that the community partners who participated in the focus groups could identify members of the public from within their community to participate.

## Procedure

Focus groups were audio-recorded and transcribed verbatim. A topic guide was developed to ensure similar topics were covered in each group, with enough flexibility to enable issues of importance to emerge. A brief presentation to demonstrate the intervention was conducted by SS or GM, and after this familiarization, the participants were asked to give their views on the intervention. A report summarizing the focus group was e-mailed to the participants for their review and validation.

## Analysis

Anonymized focus group transcripts were analyzed thematically using techniques of constant comparison [27], supported by qualitative data analysis software (NVivo 10). This involved coding and comparing emerging themes and codes within transcripts and across the dataset, looking for shared or disparate views. One transcript was double coded by KB. Four discrepancies were noted and resolved through discussion.

An additional step was taken to code the focus group data to the COM-B/TDF. This involved reading each code in NVivo and then locating the code in the list of agreed-upon mappings in the previous step. NVivo codes were mapped to the relevant COM-B components and TDF domain, with all codes mapped to at least one domain within the COM-B/TDF and some codes mapped to multiple domains. Quotes presented in the “Results” section represent examples of the identified themes.

2. Identification of the types of intervention likely to encourage timely symptom presentation among people living in socioeconomically deprived communities

The previous step reflects a behavioral diagnosis, whereby the COM-B components and TDF domains that need to be targeted in order to bring about the target behavior have been identified [24]. This diagnosis was then linked to functions through which the intervention can change behavior. The Behavior Change Wheel framework stipulates that a full range of intervention functions should be considered and provides a list of intervention functions linked to the COM-B/TDF (see Fig. 1). The nine intervention functions and the related COMB components are presented in an intervention functions matrix (see supplementary file 4). The matrix presents the links between the intervention functions and COM-B components, with the links established by an expert consensus process [24].

Intervention functions that were related to the COM-B components and TDF domains identified in the previous phase were identified and considered using the intervention function matrix [24]. For example, barriers and facilitators coded under “physical capability” may require intervention functions relating to “training” and “enablement” (see supplementary file 4). When multiple intervention functions are highlighted for consideration by the matrix, the affordability, practicability, effectiveness/cost-effectiveness, acceptability, side effects/safety, equity (APEASE) criteria are used to guide judgments for selecting the most appropriate intervention functions [24]. This step is completed by assessing the affordability, practicability, effectiveness/cost-effectiveness, acceptability, side effects/safety, and equity of each of the intervention functions in relation to the behavior. SS, GM, and KB separately applied the APEASE criteria (24) to the intervention functions, with discrepancies resolved by discussion.

3. Identification of specific intervention content to address identified barriers and facilitators to timely symptom presentation

Following identification of intervention functions, as guided by the COM-B/TDF, intervention content was identified in the form of behavior change techniques (BCTs) that would help bring about the target behavior. This is visualized as the third layer in Fig. 1 (see Intervention Functions layer of Fig. 1). BCTs that were relevant to each of the identified intervention functions were considered using the APEASE criteria (24). The full taxonomy and definitions are available elsewhere [23]. The APEASE criteria were used again by SS, KB, and GM in order to narrow down the most frequently used BCTs for each intervention function [24].

## Results

**Fig. 2:**
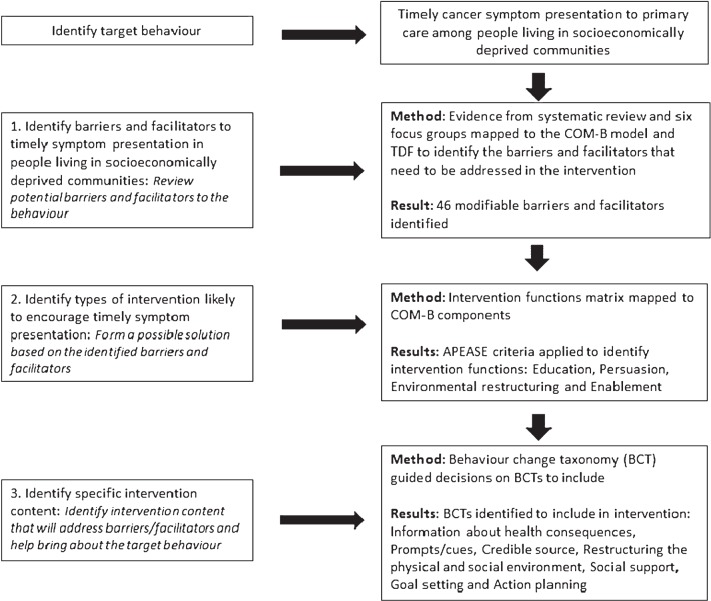
Summary of the systematic process to identify intervention content

The results relating to each of the three study objectives will be discussed separately. The intervention refinement, development process and summary of results is presented in Fig. 2.

1. Barriers and facilitators to timely symptom presentation in people living in socioeconomically deprived communities

## Systematic Review

A total of 69 barriers and facilitators were identified. When coded against the COM-B components and TDF domains, frequencies of barriers and facilitators coded were as follows: *capability*: knowledge (25); cognitive and interpersonal skills (14); memory, attention, and decision processes (12); behavioral regulation (9); physical skills (0); *opportunity*: social influences (11); environmental context and resources (19); *motivation*: reinforcement (0); emotion (18); social/professional role and identity (0); beliefs about capabilities (9); optimism (2); beliefs about consequences (11); intentions (7); goals (1).

Coding of barriers and facilitators as “modifiable” or “non-modifiable” resulted in 46 modifiable and 23 non-modifiable barriers and facilitators. The final 46 barriers and facilitators were mapped as follows: *capability*: knowledge (21); cognitive and interpersonal skills (12); memory, attention, and decision processes (11); behavioral regulation (9); physical skills (0); *opportunity*: social influences (4); environmental context and resources (3); *motivation*: reinforcement (0); emotion

(15); social/professional role and identity (0); beliefs about capabilities (8); optimism (2); beliefs about consequences (11); intentions (7); and goals (1). A list of the 46 included barriers and facilitators mapped to COM-B and TDF can be found in Table 1.

**Table 1.** Barriers and facilitators identified from systematic review mapped to COM-B model and Theoretical Domains Framework

	CAPABILITY					OPPORTUNITY		MOTIVATION							
	Physical	Psychological				Social	Physical	Reflective						Automatic	
Barriers and facilitators identified from systematic review	Physical skills	Knowledge	Cognitive and interpersonal skills	Memory, attention and decision processes	Behavioural regulation	Social influences	Environ context and resources	Beliefs about capabilities	Beliefs about consequences	Social profess role and identity	Optimism	Intentions	Goals	Reinforcement	Emotion
Perceived causes of cancer															
Knowledge about cancer screening Knowledge of what to do if or when a possible cancer symptom is detected															
Number of potential cancer symptoms															
Pain associated with potential cancer symptom															
Perceived risk of cancer															
Symptom attribution to cancer Symptom development (e.g. new symptoms appear, or reoccurrence)															
Symptom duration															
Symptom frequency															
Symptom interpretation															
Symptom knowledge															
Symptom misconceptions															
Symptom recognition															
Symptom seriousness															
Symptom severity															
Symptom type (classic v non classic)															
Symptom worsening															
Ability to articulate symptom concerns															
Concerns about wasting the doctor’s time															
Confidence in recognizing and attributing a possible cancer symptom															
Confidence in talking about a symptom															
Confidence in what to do about a possible cancer symptom and when to do it															
Embarrassment															
Anticipated regret															
Beliefs about cancer early diagnosis															
Beliefs about cancer survival outcomes															
Beliefs about treatment for cancer															
Cancer screening beliefs															
Fears about the consequences of cancer treatment															
Fatalistic cancer beliefs (e.g. there is no cure, cancer is a death sentence)															
Fear of cancer															
Fear of cancer diagnosis															
Fear of cancer diagnostic tests															
Fear of dying															
Fear of surgery for cancer															
Personal shame, stigma or blame associated with cancer															
Symptom disclosure															
Avoidance															
Denial															
Reassurance seeking from doctor															
Self-management (e.g. use of over the counter medication)															
Beliefs that the symptom will resolve on its own															
Watchful waiting															
Medical appointment that is booked for another reason leads to symptom disclosure (piggybacking)															
Intention to act on symptom (within 3 weeks)															

## Focus Groups

Focus groups took place between November 2014 and March 2015 and lasted between 64 and 82 min. In Health Board 1, the first focus group consisted of six health professionals (three general practitioners (GPs), two public health consultants, and one practice nurse), the second focus group had eight community partners, and the third had six members of the general public (four females, two males). In Health Board 2, the first focus group consisted of eight health professionals (four GPs, one practice director, one community pharmacist, one public health consultant, and one practice nurse), the second had six community partners, and the third had eight members of the public (six females, two males).

Overall, the intervention was viewed as an acceptable and useful tool for encouraging people living in socioeconomically deprived communities to engage in cancer awareness and help seeking. The focus group findings are discussed in terms of the COM-B components and TDF domain mapping (see Table 2).

**Table 2.** Illustrative quotes from focus groups aligned to COM-B model and Theoretical Domains Framework^a^

COM-B and *TDF* mapping	Description	Illustrative quote
Capability—*knowledge*	Symptoms	I think some of the symptoms can be taken as being other things. There’s one that they say, if you have heart burn a lot that can be a symptom of stomach cancer. I had no idea about that. I think for a lot of people if they had heart burn, they would just take medication to deal with the heart burn rather than them thinking it was a bigger problem. (FG2_HB1_CP)
	Risk factors	I think also there’s the fact that it’s perception that it won’t happen to them, they don’t drink and they don’t smoke. That’s a big issue. Because I don’t do X, Y and Z, I’ll not get this cancer or that cancer. I think that’s a big player in the game as well. (FG2_HB1_CP)
Capability—*cognitive and interpersonal skills, memory, attention and decision processes, behavior regulation*	Communication	A close friend of mine is a GP... she puts a lot of emphasis on what she calls the door handle diagnosis, she calls it. Someone will come to see you about something and then as they are on their way out, they’ll say, oh yes, there’s this other thing, this mole. And that’s what they’ve really come to see you about. The cold that they’ve had for a week isn’t what they’ve come to see... It’s oh, while I’m here, urn... (FG1_HB2_CP)
		Some doctors take time, don’t they? You go into them and you can talk to them, they take time to listen. And then there are other doctors, like the one I’m talking about, the first thing he does is take a prescription card and he’s writing a prescription for you before you’ve even finished telling him. You know, that’s wrong. (FG4 HB2 P)
Opportunity—*environmental context and resources*	Relationship with health professional	I’ll get on me soapbox now rather than later. One of the major problems we have in our cluster is GP appointments... And because of that, people tend to not bother, particularly with screening type things, things like that. They just... Oh, I can’t get an appointment to see the doctor when I’m bad so I’m not going to go when I’m perfectly all right, kind of attitude. (FG1_ HB2_CP)
		So they go with the one issue. And as you said, you can’t go with multiple issues. You’ve got that timeslot and you know, I’ll be fine, kind of thing, until it all blows up. (FG1_ HB2_CP)
	Competing priorities	I think a lot of our tenants, for whatever reasons, they won’t be up and be in a position where they can be ringing in or visiting a surgery at 8.30 am to get an appointment. It’s just not possible for some of them. (FG1 HB2 CP)
	Cultural factors	In many cultures, they either don’t have doctors available or they would have to pay a lot of money to see a doctor so they just assume it’s the same in this country. So it’s a lack of information about what services are available and what are on offer. (FG2_HB1_CP)
Opportunity—*social influences*	Social support	I think they would speak to some family member or friend first of all, they normally do. We still get mums coming in with sons in their thirties and forties and we still get husbands and wives come in together. Normally, the wife drags along the husband. (FG3_ HB2_HCP)
		Well, I would ask my partner, then basically, because she’s a nurse. But I also would ask my sister, who suffered cancer. I’d say "I am feeling like this". See what reaction I get and if she said well, I actually had symptoms like that, go to the doctor quickly, as soon as you can. Don’t put it off. That’s what I would do. (FG4_ HB2_P)
	Social networks	I think there’s an opportunity as well for some sort of friending scheme where people who perhaps felt a little bit, you know, vulnerable or afraid to go to see the GP by themselves, just someone that can go with them and can maybe support them, help them explain to the GP exactly what the problem is. (FG2_HB1_CP)
	Facilitated delivery of health information	It could be used with somebody in the community. I don’t think they would access it on their own (FG2_HB1_CP) It’s always going to be more effective when they are with the advisor. Listening to them talking rather than reading something. You know? Page like that come up and full of text, after something like that, a lot of people kind of think, put it together. They just can’t be bothered. Conversation is a little bit more engaging. (FG2_HB1_CP)
Motivation—*emotion*	Fearful of cancer	I think it’s the fear though perhaps because sort of on your day to day life is just ticking over. As soon as you get a diagnosis though, everything changes and I think that’s the fear of this as well that you do not want to know because things would change or potentially they do. (FG3_ HB2_HCP)
	Fear of cancer treatment	The other thing that came to mind is that people often fear treatment as well. Oh, chemotherapy, I’m going to lose my hair. That’s the first thing that they think of, losing their hair, rather than curing the cancer. So that sort of thing might help. (FG5 HB1 HCP)
	Gender differences	Because women are giving birth, we are more sort of, you know? I think sometimes men put off going to the doctors, don’t they? Because they are embarrassed. (FG4_HB2_P)
	Cultural beliefs	It’s always been classed as a private, you don’t talk about anything down there. And it’s still a culture. Especially British people, still have that stiff upper lip, don’t talk about that. (FG5_ HB1_HCP)
Motivation—*beliefs about capabilities and beliefs about consequences*	Negative cancer beliefs	Cancer is like a stigma... and they think it’s a death sentence, it’s not. Cancer has become a chronic disease now, it’s like diabetes and other things, we’re talking about 10 or 20 years survival rates. (FG3_ HB2_HCP)
	Self-efficacy	And people’s expectations of their own health and the health of their family depending on where they live. So living in an area where there is relatively low life expectancy, high level of premature mortality, everyone that you know has got a chronic condition, that’s probably what you expect yourself. So maybe there is a lack of motivation to make changes because it just seems, it’s that external levels of control, you just think well, these things are outside of my control. That’s how I’m going to end up. I think it’s very difficult for people to change if you ignore the context in which they live their lives. (FG5_HB1_HCP)
		If they have a symptom of something, they will try and minimize it. Oh, have I got blood, losing blood, it’s from something else. They always find another reason of what it could be because they don’t want to confront the fact that it could be that, try to instill a bit of confidence in them. Go and get yourself checked out, you know? (FG2_ HB1_CP)

a Insertions to clarify topic content are denoted by square brackets. The removal of irrelevant information within the quotes is denoted by "....". Focus group characteristics are provided at the end of quotes: focus groups (FG) are numbered 1-6, the location of the focus group is included as either Health Board 1 (HB1) or Health Board 2 (HB2), and the focus group members were either general public (P), community partners (CP) or healthcare professionals (HCP)

## Capability

### Knowledge

A lack of knowledge in the community was discussed in relation to cancer symptoms and risk factors. Lack of knowledge was thought to influence symptom interpretation and decisions about when and where to seek help. The non-specificity of many cancer symptoms was described as making it difficult for people to attribute symptoms to cancer. This could sometimes lead to minimizing symptoms and attribution to benign or everyday physical sensations. Fatalism and fear were perceived to stem from poor knowledge, with participants discussing how people in their community held outdated views on cancer outcomes. Crisis point care in the community was discussed, with this describing the act of

All items listed can be a barrier or facilitator; the directionality varies depending on individuals and circumstances; therefore, the directionality is not specified

presenting with signs or symptoms that are progressed to an advanced stage. Crisis point care was perceived to be a consequence of lack of capability, with people in the community sometimes waiting until symptoms reached a point where they impacted on day-to-day life before seeking help.

#### Cognitive and Interpersonal Skills, Memory, Attention and Decision Processes, and Behavior Regulation

Skills to appraise symptoms and communicate concerns, as well as decisions to seek help, were perceived to be influenced by an individual’s psychological capability. Engaging and communicating with healthcare professionals was perceived to be difficult for many people in the community. This perceived difficulty was described as being influenced by lack of confidence in discussing symptoms, the preparedness of the GP to listen to symptom concerns, and previous experiences. When people think about presenting with symptoms, they draw on previous decisions they have made, previous symptom experiences, and previous experiences with doctors. Therefore, the three TDF domains of cognitive and interpersonal skills, memory, attention and decision processes, and behavior regulation are closely linked.

## Opportunity

### Physical Opportunity (Environmental Context and Resources)

Service barriers, such as difficulty getting appointments and problems with appointment booking processes, were frequently mentioned. Consistency of GP was considered important, with lack of continuity of care making it harder for people to voice health concerns. Length of time in primary care appointments was described as a barrier to discussion about health concerns, with the duration of appointments not long enough for patients to explain their health concerns, leading to feelings of being rushed and pressured.

Competing priorities and life commitments were also discussed. It was acknowledged that people living in deprived communities often have commitments and concerns, such as family, housing, or people who depend on them, limiting the opportunities to look after their own health. These competing priorities and commitments were perceived as compounding the issues surrounding booking processes, leading people to neglect their own health. Long-term health was perceived not to be a priority for many people in the local community. The cultural diversity of the community was also discussed, with reflection that people who have moved to the local community from other countries were not aware that primary care could be a place to discuss health concerns.

#### Social Opportunity (Social Influences)

Support from family and friends was perceived to encourage people to seek help, and those without social support were described as less likely to present. Social support was perceived to facilitate many aspects of help seeking, from symptom appraisal and making the decision to seek help to discussing symptom concerns with health professionals. It was thought that people in the community would often turn to family members, friends or elders to talk about health concerns. Social circles were also an opportunity to acquire knowledge about cancer symptoms, treatment, and curability. Participants discussed how people in their community would often draw on experiences of family and friends when considering aspects of their own health. Building relationships with community partners or other people in the community was suggested as a way to help people become more aware and engaged in their health by making it easier to disclose health issues. It was discussed how people in the local community find it easier to discuss health with those they trust and may be more receptive to the advice and support provided by the trusted person (e.g., friend, family, or elder).

When access to health information and the delivery of the health check was discussed, the participants thought people in their community may not have opportunities to access information on their own. For example, people may not know where to look for information or would not have access or the skills to access online health information. It was suggested that community partners were trusted people who could engage people in a conversation about their health and provide support in the form of the health check intervention.

## Motivation

### Emotion (Automatic Motivation)

Emotions were considered to influence motivation to present in the presence of symptoms. Automatic emotional responses such as fear of cancer were thought to influence people in the community on different levels, whether through general fear of being diagnosed with a life-threatening illness, fear of cancer itself, or fear of cancer treatment. Embarrassment was suggested as an emotional barrier that was particularly relevant for signs and symptoms originating from intimate areas of the body and was thought to reflect British cultural attitudes of embarrassment and maintaining a “stiff upper lip.” Gender differences were also highlighted, with men thought to be less motivated and more inclined to delay presentation than women were.

#### Beliefs about Capabilities and Beliefs about Consequences (Reflective Motivation)

Discussion centered on beliefs about early diagnosis, treatment, and the curability of cancer. Concerns were expressed about prevailing negative beliefs about cancer within the local community, which were described as influencing people’s motivation to engage with preventive or protective health behaviors. Participants discussed how cancer is perceived as beyond the individual’s control, non-treatable, and a death sentence. These beliefs were thought to stem from experience of cancer in family or friends and could reduce people’s motivation to engage with their health. Participants felt that much could be gained by encouraging positive beliefs, for example, by emphasizing current treatment successes and benefits of early diagnosis. It was also discussed how people living in socioeconomically deprived populations may take longer to act on symptoms, instead hoping the symptom will resolve by itself and only seeking help when the symptom can no longer be ignored. Lack of confidence in recognizing and attributing symptoms was also raised as a barrier to presentation.

2. Types of intervention likely to encourage timely symptom presentation

Mapping the COM-B components to the intervention function matrix identified that all of the intervention functions needed to be considered. Applying the APEASE criteria (24) led to the identification of the following intervention functions for inclusion: *education, persuasion, training, environmental restructuring*, and *enablement* (see Table 3). *Education* formed a central part of the intervention, involving the provision of information aimed at increasing knowledge about symptoms and what to do in their presence. *Persuasion* was selected to reflect the intervention’s potential to encourage and advise people who are currently experiencing symptoms to see their GP promptly. Persuasive language could be used to emphasize the importance of presenting and the health benefits of early presentation.

*Training* was selected to reflect that timely symptom presentation could be achieved by training intervention users to create action plans in response to potential cancer symptoms, enabling users to act on symptoms that they may experience. *Environmental restructuring* was selected because the intervention requires the physical and social context of the individual to be altered in order for early symptom presentation to be achieved. Taking the intervention into the community and delivering it in non-traditional medical settings is one way of achieving this. Finally, *enablement* was selected because increasing means and reducing barriers to presentation is important in order for early symptom presentation to be achieved. Modeling was considered impractical and unlikely to meet the affordable and effectiveness/cost-effectiveness aspects of APEASE (24). Incentivisation was not applicable due to financial incentives for such an intervention deemed

**Table 3.** Selection of intervention functions based on the APEASE criteria

Intervention functions	Does the intervention function meet the APEASE criteria in the context of presenting to the GP with a possible cancer symptom?					
	Affordability	Practicability	Effectiveness/cost-effectiveness	Acceptability	Side effects/safety	Equality
Education^a^						
Persuasion^a^						
Incentivisation						
Coercion^a^						
Training^a^						
Restriction						
Environmental restructuring^a^						
Modelling^a^						
Enablement^a^						

unaffordable and unacceptable to professional stakeholders. Coercion and restriction were also considered to be unacceptable to users and providers.

3. Specific intervention content to address identified barriers and facilitators to timely symptom presentation

The APEASE criteria were applied to the most frequently used BCTs [24] for each of the identified intervention functions (see supplementary file 5). A summary of intervention functions and BCTs that were selected to address the barriers and facilitators to the target behavior is presented in Table 4, alongside examples of strategies that describe how the BCTs will be delivered in the modified intervention. These examples cover changes to the touchscreen questionnaire and the individualized results. This table represents a summary of all of the phases of the Behavior Change Wheel and is the culmination of all of the phases of work in the current study.

**Table 4.** Summary of COM-B model, Theoretical Domains Framework, intervention functions, behavior change techniques, and proposed intervention content

COM-B and TDF	Intervention functions	Behavior change techniques selected	Intervention strategy (examples of application within the intervention)
Influencing capability			
Knowledge	Education, persuasion	Information about health consequences	• Questions and results included for 12 symptoms which reflect current guidelines
			• Results emphasize the benefits of early presentation
			• Results highlight improvements in cancer treatment to reduce negative cancer beliefs
Influencing opportunity			
Environmental context and resources	Environmental restructuring, enablement	Restructuring the physical environment	• The intervention is delivered in a variety of non-medical community settings
Environmental context and resources	Education, environmental restructuring	Prompts/cues	• Questions ask whether people have received and taken up relevant screening invitations (based on age and gender)
			• Results encourage participants to look for lumps when in the shower. This will lead to the shower acting as prompt/cue for checking for lumps.
Social influences	Environmental restructuring, enablement	Restructuring the social environment	• Results encourage people to tell others about symptom concerns
			• A lay advisor will be available to do the health check with the individual and will build a rapport with the participant to facilitate delivery of the health information
Social influences	Persuasion	Credible source	• The intervention is associated with Tenovus Cancer Care as a credible source
			• Intervention is delivered by a lay advisor as a trusted source of information and support
Social influences	Enablement	Social support (unspecified)	• Results encourage people to take someone with them to primary care appointments
			• Results provide information and advice for making a primary care appointment
			• Results emphasize GP support and availability
Influencing motivation			
Goals	Enablement	Goal setting (behavior)	• The goal of timely presentation with potential cancer symptoms is presented throughout the intervention results
			• People who receive “red results” receive information telling them to seek medical advice
			• Those who receive green results are presented with information about the symptom to enable them to seek help if they experience the symptom in the future
Goals	Enablement	Goal setting (outcome)	• Participants set a goal to present to primary care with potential cancer symptoms. This is a positive outcome of increased symptom knowledge and also represents knowledge of the importance of early diagnosis
Intentions	Enablement	Action planning	• Action planning will be prompted after the results by people completing the statement in the results printout “If I notice a symptom, I will go and see my ____ within ______ of noticing the symptom”
Beliefs about	Education, persuasion	Information about health	• Results emphasize the benefits of early presentation
consequences		consequences	• Results highlight improvements in cancer treatment to reduce negative cancer beliefs

Provision of information about health consequences in the intervention could educate people to seek help and act as persuasion in the presence of symptoms (see Table 4). Emphasizing the benefits of early presentation and improvements in cancer treatments may encourage people to act earlier in the presence of symptoms. Information on symptoms that may indicate cancer and when to act on them is crucial content for the health check and could act as a prompt or cue to action, which involves introducing or defining environments so that people are prompted or cued to the behavior (see Table 4). Goal setting and action planning were also identified as valuable BCTs and emphasize the importance of acting in the present but also planning for the future. This is important because many people who complete the intervention may not be currently experiencing symptoms but may go on to do so (see Table 4).

Credible source relates to presenting verbal or visual information for or against the behavior by a credible source, which will be the health check advisor on behalf of Tenovus Cancer Care (see Table 4). The health check advisor will help restructure the social environment by being on hand to facilitate the individual to go through the intervention and encourage them to act on their own personalized results (see Table 4). Other social support strategies will involve emphasizing facilitators to presentation such as social networks and GP preparedness to listen to symptom concerns. Restructuring the physical environment is a change in the physical environment in order to facilitate performance of the behavior [24]. By restructuring the physical environment and taking the intervention to people in community settings, barriers to discussing health concerns, such as appointment processes, may be reduced (see Table 4). Engaging people within community settings may facilitate discussions about health and act as a springboard from which people can be encouraged by the lay advisor to seek advice from a healthcare professional.

The refined health check contains 26 questions in three domains: about you (7), your lifestyle (5), and your health (14) (see supplementary file 2 for full list of questions). Symptom questions and results have been refined to reflect the latest clinical guidelines, with lifestyle and personal history questions and responses also refined to reflect current clinical guidelines for the referral and recognition of suspected cancer in the UK (https://www.nice.org.uk/guidance/ng12?unlid=49677604320162173631). Questions and results about screening has also been developed to reflect the importance of discussing ways to manage cancer risk and engagement with screening services.

## Discussion

The Behavior Change Wheel [22] and its constituent elements were applied to refine and develop an intervention that aims to encourage timely symptom presentation to primary care among people living in socioeconomically deprived communities. To our knowledge, the current study is the first to apply the Behavior Change Wheel in this context. This systematic approach provided insights into cancer symptom awareness, help-seeking behaviors, and barriers and facilitators to early presentation in people living in socioeconomically deprived communities. The study also demonstrates how primary and secondary data can be applied to the Behavior Change Wheel for intervention development and refinement. The COM-B and TDF [22] were used to map important barriers and facilitators relating to presentation to primary care with possible cancer symptoms in socioeconomically deprived communities and formed the theoretical basis for refining the intervention. The behavior change taxonomy was then drawn upon to specify the intervention content, enabling barriers and facilitators to be targeted in order to encourage earlier presentation. Primary qualitative data collection added to the secondary systematic review data by enabling exploration of identified barriers and facilitators.

Findings suggest that capability, opportunity, and motivation play an important role in cancer awareness and help seeking for potential cancer symptoms in people living in socioeconomically deprived populations, and that these constructs are not independent, and all contribute to decisions surrounding presentation. This was evident in the overlapping influence of many of the constructs in our systematic review and focus group analysis. Findings were incorporated into an intervention to increase cancer awareness and encourage help seeking; 4 intervention functions (education, persuasion, environmental restructuring, and enablement) were identified to address 46 modifiable barriers and facilitators in an affordable, practical, and acceptable way. Behavior change techniques aligned to these four functions were then selected in order to address the barriers and facilitators to early symptom presentation for people living in socioeconomically deprived communities.

Previous studies have highlighted barriers to symptom presentation among socioeconomically deprived populations, including fatalism, fear of diagnosis, and concerns about wasting the doctor’s time [6, 11, 14, 15]. The present study adds to the existing literature by suggesting that social networks and systems influence barriers and facilitators to presentation in people living in socioeconomically deprived communities. The influence of social networks suggests that there is a lay system of healthcare, whereby people seek help and advice from friends or family [28, 29]. In such cases, the health literacy of friends and family is regarded as an “asset” [30] and is drawn upon by people with lower health literacy. This willingness to seek help from others and engaging with health can be viewed positively. However, if the confidante lacks knowledge or has had a negative personal experience with the health system, this could potentially lead to erroneous advice that could delay presentation. The use of a lay system of healthcare highlights the importance of improving community levels of cancer awareness and dispelling negative beliefs about cancer.

Opinions were expressed in the focus groups that some community members may possess outdated or incorrect information about cancer symptoms, risk factors, and treatments. Recognizing the public image of cancer has been identified as an important first step for the delivery of cancer messages [31]. Understanding existing perceptions is important to be able to provide information that aims to modify perceptions through beliefs about consequences. Information that emphasizes early diagnosis and related benefits may encourage people to act in the presence of symptoms, with this echoed in recommendations for future intervention research in a recent review of socioeconomic differences in responses to possible cancer symptoms [7].

Information on symptoms that may indicate cancer and when to act on them is central to the current intervention and could act as a prompt or cue to action, with previous studies suggesting that high levels of symptom awareness are associated with earlier presentation [6, 14, 19]. This information will be useful to people who are currently experiencing symptoms, as it will enable them to seek help following the intervention but will also be useful for those who experience symptoms in the future. Action planning [32] could also be used to help override the automatic negative associations of cancer as a death sentence and other fatalistic beliefs.

Recommendations for intervention delivery were also gained in the current study. Preferences were expressed for facilitated delivery of the health check in community settings, for example, by a trained lay advisor [33]. It may therefore be worthwhile training community partners to deliver the intervention. Doing so could increase the number of completed interventions due to increased access and confidence to discuss health with someone familiar, as opposed to a perceived outsider. A trained community member could act as a credible source, and delivery by such a person in community settings will restructure the physical environment. Both of these recommendations are examples of understanding the needs of the community and demonstrate the importance of embedding interventions in local needs and preferences, as outlined in the Medical Research Council complex intervention guidelines [20].

## Strengths and Limitations

The study was underpinned by a strong theoretical base and has demonstrated that the COM-B Model and TDF can be systematically applied to the identification of barriers and facilitators to symptom presentation to primary care in people living in socioeconomically deprived communities. This study has also demonstrated how focus groups (primary data) and a systematic review (secondary data) can be applied to the Behavior Change Wheel to guide intervention development and refinement. The use of qualitative methods enabled in-depth exploration of barriers and facilitators to presentation, in addition to other contextual factors arising during the focus groups. This led to identification of other relevant ideas for consideration, such as intervention delivery recommendations. The systematic review therefore proved beneficial for ensuring the identification of existing barriers and facilitators in the literature, with the focus groups enabling deeper exploration of these, in addition to the opportunity to identify any new barriers and facilitators and to explore other relevant contextual factors that could enhance the intervention.

The Behavior Change Wheel triangulates the findings from these sources and enables intervention content to be developed that takes into account the barriers and facilitators to the target behavior, in the target population. The systematic approach afforded by the Behavior Change Wheel ensures that intervention developers consider the target audience at every stage of development and have a clear set of steps to follow to ensure that the intervention reflects the needs of the target audience. Although subjectivity is a potential problem in the process of developing interventions, this was minimized by having three team members involved in the Behavior Change Wheel process. Future research could go beyond this and involve stakeholders in processes, such as applying the APEASE criteria, to try to gain wider opinions and judgments.

The focus group analysis was conducted inductively, with themes identified from this approach then mapped to the COM-B/TDF codes identified from the systematic review. This approach was chosen to ensure that all themes within the data were identified and not restricted to the COM-B components and TDF domains. However, when the focus group codes were mapped to the COM-B/TDF, all codes were aligned to at least one COM-B component and TDF domain. This suggests that future studies may benefit from deductively mapping qualitative findings to COM-B components and TDF domains.

The focus groups were completed in two Welsh Health Boards, and therefore results may not be generalizable to people outside of these areas. However, previous studies have shown similar levels of cancer knowledge, beliefs, and barriers in Wales, England and Northern Ireland [6]; hence, the findings may be applicable and transferable to other UK settings. The current study provides an approach to applying the Behavior Change Wheel to intervention development that draws upon both primary and secondary data sources to create an intervention based on empirical evidence and embedded in the needs and preferences of the target users. The findings may therefore be transferable to other studies of intervention development and refinement. Transparency in the reporting of intervention details is important for progressing the field of intervention development and behavior change [34]; therefore, the health check questionnaire, results, and accompanying manual for intervention delivery will be published in a future paper alongside the evaluation.

The opportunistic sampling methods also limit representativeness. This kind of sampling can lead to clustering of participants among certain groups of individuals, such as health professionals who have a particular interest in cancer awareness or who are particularly outspoken in their professional groups, rather than those with no special interest who are perhaps more representative of the health profession as a whole.

## Conclusions

The Behavior Change Wheel enabled intervention content to be developed and refined using a theoretically driven method and led to specific BCTs being incorporated in the intervention that will maximize the likelihood of the target behavior being carried out [24]. The Behavior Change Wheel can be used to triangulate the findings from primary (focus groups) and secondary (systematic review) data sources and enables intervention content to be developed that takes into account the barriers and facilitators to the target behavior. Social networks were found to be prominent in shaping health-related knowledge and actions for people from socioeconomically deprived communities. A community-based intervention facilitated by a trained community member may prove effective at engaging hard-to-reach groups and maximizing intervention usability, acceptability, reach, and feasibility. The intervention was refined using rigorous theoretically based methods and will be evaluated for its influence on cancer symptom awareness and help-seeking behavior in socioeconomically deprived communities.

## Supplementary Material

Supplementary File 1

Supplementary File 2

Supplementary File 3

Supplementary File 4

Supplementary File 5
