# Supplementary figures and images for: Development of a Behavior Change Intervention to Encourage Timely Cancer Symptom Presentation Among People Living in Deprived Communities Using the Behavior Change Wheel
Source: Ann Behav Med. 2017 Dec 13;52(6):474–88. doi: 10.1007/s12160-016-9849-x (PMC6367899; doi:10.1007/s12160-016-9849-x)

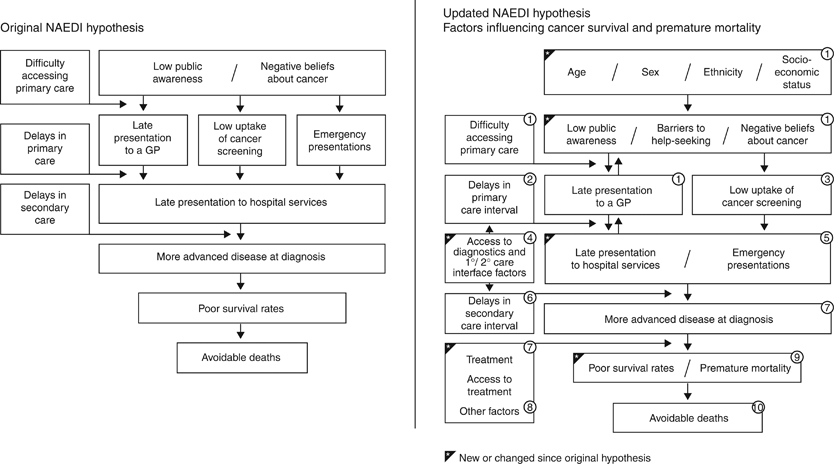


Supplementary file 1. Updated National Awareness and Early Diagnosis Initiative pathway (8)

Supplement: Supplementary File 1 [file s12160-016-9849-x_supplementary_file_1.docx]
