# Supplementary material for: Development of a Behavior Change Intervention to Encourage Timely Cancer Symptom Presentation Among People Living in Deprived Communities Using the Behavior Change Wheel
Source: Ann Behav Med. 2017 Dec 13;52(6):474–88. doi: 10.1007/s12160-016-9849-x (PMC6367899; doi:10.1007/s12160-016-9849-x)
Supplement: Supplementary File 2 [file s12160-016-9849-x_supplementary_file_2.docx]

Supplementary file 2: Question content of original and modified health check

Existing health check: 30 questions in three domains:

- Your History (four questions)
  1. Have you ever been diagnosed with cancer?
  2. Are there two or more close relatives on the same side of your family who have had cancer?
  3. Do you have any relatives who have been under the age of 45 when diagnosed with cancer?
  4. Please give you height and weight
- Your Lifestyle (12 questions)
  1. On average how many days a week do you exercise for half an hour or more?
  2. Do you smoke? How many?
  3. Do you drink alcohol? How much?
  4. How often do you normally eat fruit?
  5. How often do you normally eat vegetables?
  6. How often do you normally eat fatty foods?
  7. How often do you normally eat red meat?
  8. How often do you normally eat cakes, biscuits and sweets?
  9. Have you ever suffered sunburn?
  10. Do you use sun cream when you are out in the sun? What factor sun cream do you use?
  11. Do you cover up when you are out in the sun?
  12. Do you use sun beds?
- Your Health (14 questions)
  1. Have you noticed any change in your appetite in the last 12 months?
  2. Have you been losing weight without trying to?
  3. Have you noticed any changes in your bowel motions in the last 12 months?
  4. Have you noticed blood in your bowel motion in the last 12 months?
  5. Have you noticed any changes in the appearance of your skin in the last 12 months?
  6. Do you have any moles?
  7. Do you have a regular persistent cough?
  8. Do you bring up phlegm when you cough? Is your phlegm ever blood stained?
  9. Do you regularly check your body for lumps?
  10. Have noticed any unusual lumps in the last 12 months?

*Female only*: 11.Have you gone through, or are you going through the menopause?

12. During the last 12 months, have you had pain or bleeding during or after sex?

13. During the last 12 months have your periods been irregular?

14. During the last 12 months have you experienced bleeding in between your periods?

*Male only*: 11. How many times do you need to get up at night to urinate?

12. Do you have any pain or discomfort when urinating?

13. When urinating do you have any problem with flow?

14. Do you have difficulties starting or finishing urinating?

15. Do you have any other problems when urinating?

Modified health check: 26 questions in three domains:

- About You (7)
  1. Have you ever been diagnosed with cancer?
  2. Are there two or more close relatives on the same side of your family who have had cancer?
  3. Do you have any relatives who have been under the age of 50 when diagnosed with cancer?
  4. Please give your height and weight
  5. Have you been invited for a cervical smear test? Did you attend?
  6. Have you been invited for breast screening? Did you attend?
  7. Have you been invited for bowel screening? Did you return your kit?
- Your Lifestyle (5)
  1. Do you smoke? How many?
  2. Are you exposed to another person’s smoke?
  3. Do you drink alcohol? How much?
  4. On average how many days a week do you exercise for half an hour or more?
  5. How often do you eat 5 portions of fruit and vegetables a day?
- Your Health (14)
  1. Do you have a cough that won’t go away? Do you bring up blood when you cough?
  2. Have you noticed any unusual lumps on your body?
  3. Have you noticed a change in how your skin looks?
  4. Do you have a sore or ulcer in your mouth that will not heal?
  5. Have you noticed a change in your poo?
  6. Have you noticed any blood in your poo?
  7. Do you have any problems when peeing?
  8. Do you have any unexplained bleeding?
  9. Do you have difficulty swallowing?
  10. Have you been losing weight without trying to?
  11. On most days, do you feel bloated?
  12. Have you noticed any unexplained change in your appetite?
  13. Do you feel tired most of the time?
  14. Do you have an unexplained pain that won’t go away?
