# Supplementary material for: Development of a Behavior Change Intervention to Encourage Timely Cancer Symptom Presentation Among People Living in Deprived Communities Using the Behavior Change Wheel
Source: Ann Behav Med. 2017 Dec 13;52(6):474–88. doi: 10.1007/s12160-016-9849-x (PMC6367899; doi:10.1007/s12160-016-9849-x)
Supplement: Supplementary File 3 [file s12160-016-9849-x_supplementary_file_3.docx]

Supplementary file 3. Focus group topic guide. Please note, the topic guide was generic for the three types of focus group, with a slight change of wording depending on the specific group.

**Part 1: Intervention content**

1. **Influences on cancer awareness and**

**help-seeking in the community**

**1. Is health a concern for you? Is health a concern for people in your community?**

Is cancer a concern for you? Is cancer a concern for people who live in your community?

How do you think cancer compares to other diseases e.g. diabetes or heart disease?

What do you think about the treatments for cancer?

**Activity (write responses to the following question on individual cards):** Can you think of any symptoms of cancer? *Can you remember how you found out that these were symptoms of cancer?*

**2. What would you do if you noticed a change in your body that you think might be cancer?** Would you tell anyone? Why would you tell this person? What do you think they would say?

What would influence you in deciding what to do?

Has anyone ever asked you for advice about a symptom? What advice did you give? Why do you think they came to you for advice?

What do you think other people who live in your community would do if they noticed a symptom that they think might be cancer?

Do you ever consciously look out for symptoms of cancer?

**3. Thinking about health care professionals e.g. doctors, nurses, pharmacists etc, who would you go to first if you thought you had a symptom of cancer?**

**4. Is there anything that would stop you going to see your doctor with a symptom of cancer?**

If you had a symptom that you think might be cancer, what would you say to your doctor? Would you go just for this symptom or for something else and then tell them about this symptom?

What might encourage you to go?

Probes can be barriers from BCW: knowledge, confidence, fear etc i.e. Do you think things like fear would stop you going to the doctor with a symptom of cancer?

**5. If you needed to go to the doctor, how easy would it be for you to go and see them?**

What is it like getting an appointment? How easy do you find it to talk to the doctor? Is distance/travel a problem? Do you feel confident talking about symptoms with your doctor?

Are there other things that get in the way when going to see the doctor? Probe – attitudes, awareness, beliefs… i.e. What about your relationship with the doctor?

Do your family members or friends influence your decision to go and see the doctor? Do you take anyone with you when you go to the doctors? Has experience of cancer in friends or family influenced your thoughts about cancer?

What are the most important things influencing your decision to see the doctor?

**Activity:** Look at the symptoms on the individual cards (previously generated from earlier activity). Ask participants to rank the symptoms in terms of how quickly they would make an appointment with their doctor.

**B. the Tenovus health check**

- **Now let’s talk about the Tenovus Health Check……..** Demonstration of the health check by the researcher (orientating to its cancer symptom awareness / early diagnosis functions).

**1. What do you like about the Health Check? What do you dislike?**

Pros and cons of content, format.

What do you think about the information section at the end?

**2. What do you think are the benefits of using the Health Check?**

Do you think the health check will help you to know more about the symptoms of cancer? Do you think the Health Check will help you to know what to do if you had a symptom of cancer?

If someone offered to do the Health Check with you, what would you do/say? What about other people in your community?

What do you think about the questions in the health check about cancer symptoms? (Orientate and remind participants about which questions these are)

Can you think of any problems with using the Health Check?

**3. Do you think anything else could be included in the Health Check to encourage people to go to their doctor quickly?**

For example, inclusion of additional cancer symptoms, links to age-appropriate screening recommendations, use of positive messaging to change beliefs, social encouragement/support, action planning (define).

Can you think of anything else that you would like to see included in the Health Check?

**4. Can you think of places where would it be good to have the health check?**

For example GP practice, Tenovus mobile unit, community centre, libraries, local pharmacy, sports clubs?

Probe issues of confidentiality, embarrassment, stigma etc

Can you think of ways we could advertise the health check?

What do you think about having the health check online?

What do you think about going through the Health check with a trained health check advisor? Who would you be most comfortable doing the health check with?

**5. Have you done anything like the Health Check before?**

How does it compare?
