# Supplementary material for: Development of a Behavior Change Intervention to Encourage Timely Cancer Symptom Presentation Among People Living in Deprived Communities Using the Behavior Change Wheel
Source: Ann Behav Med. 2017 Dec 13;52(6):474–88. doi: 10.1007/s12160-016-9849-x (PMC6367899; doi:10.1007/s12160-016-9849-x)
Supplement: Supplementary File 4 [file s12160-016-9849-x_supplementary_file_4.docx]

Supplementary file 4. Intervention Function Matrix [26]

| **Model of Behaviour: sources** | **Intervention Functions** | | | | | | | | |
| --- | --- | --- | --- | --- | --- | --- | --- | --- | --- |
|  | Education | Persuasion | Incentivisation | Coercion | Training | Restriction | Environmental restructuring | Modelling | Enablement |
| Physical Capability |  |  |  |  |  |  |  |  |  |
| Psychological Capability |  |  |  |  |  |  |  |  |  |
| Physical Opportunity |  |  |  |  |  |  |  |  |  |
| Social Opportunity |  |  |  |  |  |  |  |  |  |
| Automatic Motivation |  |  |  |  |  |  |  |  |  |
| Reflective Motivation |  |  |  |  |  |  |  |  |  |
