# Supplementary material for: Development of a Behavior Change Intervention to Encourage Timely Cancer Symptom Presentation Among People Living in Deprived Communities Using the Behavior Change Wheel
Source: Ann Behav Med. 2017 Dec 13;52(6):474–88. doi: 10.1007/s12160-016-9849-x (PMC6367899; doi:10.1007/s12160-016-9849-x)
Supplement: Supplementary File 5 [file s12160-016-9849-x_supplementary_file_5.docx]

Supplementary file 5. Selection of behaviour change techniques based on APEASE criteria

| **Candidate intervention functions** | **Individual behaviour change techniques*** | **Does the BCT meet the APEASE criteria (affordability, practicability, effectiveness/cost-effectiveness, acceptability, side effects/safety, equity)?** |
| --- | --- | --- |
| **Education** | - Information about social and environmental consequences - Information about health consequences - Feedback on behaviour - Feedback on outcome(s) of the behaviour - Prompts/cues - Self-monitoring of behaviour | - Not relevant in this context - Yes - Not relevant in this context - Not practical - Yes - Not practical |
| **Persuasion** | - Credible source - Information about social and environmental consequences - Information about health consequences - Feedback on behaviour - Feedback on outcome(s) of the behaviour | - Yes - Not relevant in this context - Yes - Not relevant in this context - Not practical |
| **Environmental restructuring** | - Adding objects to the environment - Prompts/cues - Restructuring the physical environment - Restructuring of social environment | - Not relevant in this context - Yes - Yes - Yes |
| **Enablement** | - Social support (unspecified) - Social support (practical) - Goal setting (behaviour) - Goal setting (outcome) - Adding objects to the environment - Problem solving - Action planning - Self-monitoring of behaviour - Restructuring the physical environment - Review behaviour goal(s) - Review outcome goal(s) | - Yes - Not practical - Yes (if have a symptom) - Yes (If have a symptom) - Not relevant in this context - Not practical - Yes - Not practical - Yes - Not affordable or practical - Not affordable or practical |
| **BCTs selected** | Information about health consequences  Prompts/cues  Credible source  Restructuring the physical environment  Restructuring the social environment  Social support (unspecified)  Goal setting (behaviour)  Goal setting (outcome)  Action planning | |

* Most frequent BCTs for each intervention function
